# Bacterial alginate metabolism: an important pathway for bioconversion of brown algae

**DOI:** 10.1186/s13068-021-02007-8

**Published:** 2021-07-18

**Authors:** Lanzeng Zhang, Xue Li, Xiyue Zhang, Yingjie Li, Lushan Wang

**Affiliations:** State Key Laboratory of Microbial Technology, Shandong University, Qingdao, 266237 China

**Keywords:** Alginate, Alginate lyase, Alginate-utilising systems, Application of alginate lyases, Metabolic engineering

## Abstract

Brown macroalgae have attracted great attention as an alternative feedstock for biorefining. Although direct conversion of ethanol from alginates (major components of brown macroalgae cell walls) is not amenable for industrial production, significant progress has been made not only on enzymes involved in alginate degradation, but also on metabolic pathways for biorefining at the laboratory level. In this article, we summarise recent advances on four aspects: alginate, alginate lyases, different alginate-degrading systems, and application of alginate lyases and associated pathways. This knowledge will likely inspire sustainable solutions for further application of both alginate lyases and their associated pathways.

**Supplementary Information:**

The online version contains supplementary material available at 10.1186/s13068-021-02007-8.

## Background

Based on the medium-variant projection, the world’s population of 7.7 billion in mid-2019 is estimated to reach around 8.5 billion in 2030, 9.7 billion in 2050, and 10.9 billion in 2100 [1]. Therefore, it is urgent to find alternative renewable energy sources. Marine macroalgae (seaweeds), including green, red and brown macroalgae, have attracted attention as an alternative feedstock for the production of both biofuels and chemicals due to their high content of suitable polysaccharides, large-scale cultivation, and no requirement for arable land, freshwater or fertiliser [2]. Compared with green macroalgae that are mainly composed of relatively easily fermentable glucans, utilisation of red and brown macroalgae is more difficult because some members of their major polysaccharides are not easily fermented, such as 3,6-anhydro-l-galactose in red macroalgae and alginate in brown macroalgae. In recent decades, countless enzymes have been identified for degradation of the major carbohydrates in red and brown macroalgae, and the seaweed-derived products are widely used in different fields, as summarised in several reviews [3–6]. For example, alginates show a number of applications in biomedical science and engineering due to their favourable properties, such as wound dressing, drug delivery, tissue engineering, and other applications [4]. In addition, the degradation products of alginate polymers—alginate oligosaccharides (AOS) have also shown health beneficial effects, such as immunomodulatory, antimicrobial, antioxidant, and other activities [3]. Moreover, advances in metabolic engineering allow us to convert these major carbohydrates to ethanol by expressing a complete biosynthesis pathway in a single microbial host [7, 8].

In this review, we discuss recent progress in the field, with emphasis on bacterial alginate degradation in vitro and in vivo, and particular focus on alginate, alginate lyases, microbial strategies for alginate degradation, and application of alginate lyases and alginate-degrading pathways.

## Alginate

**Fig. 1:**
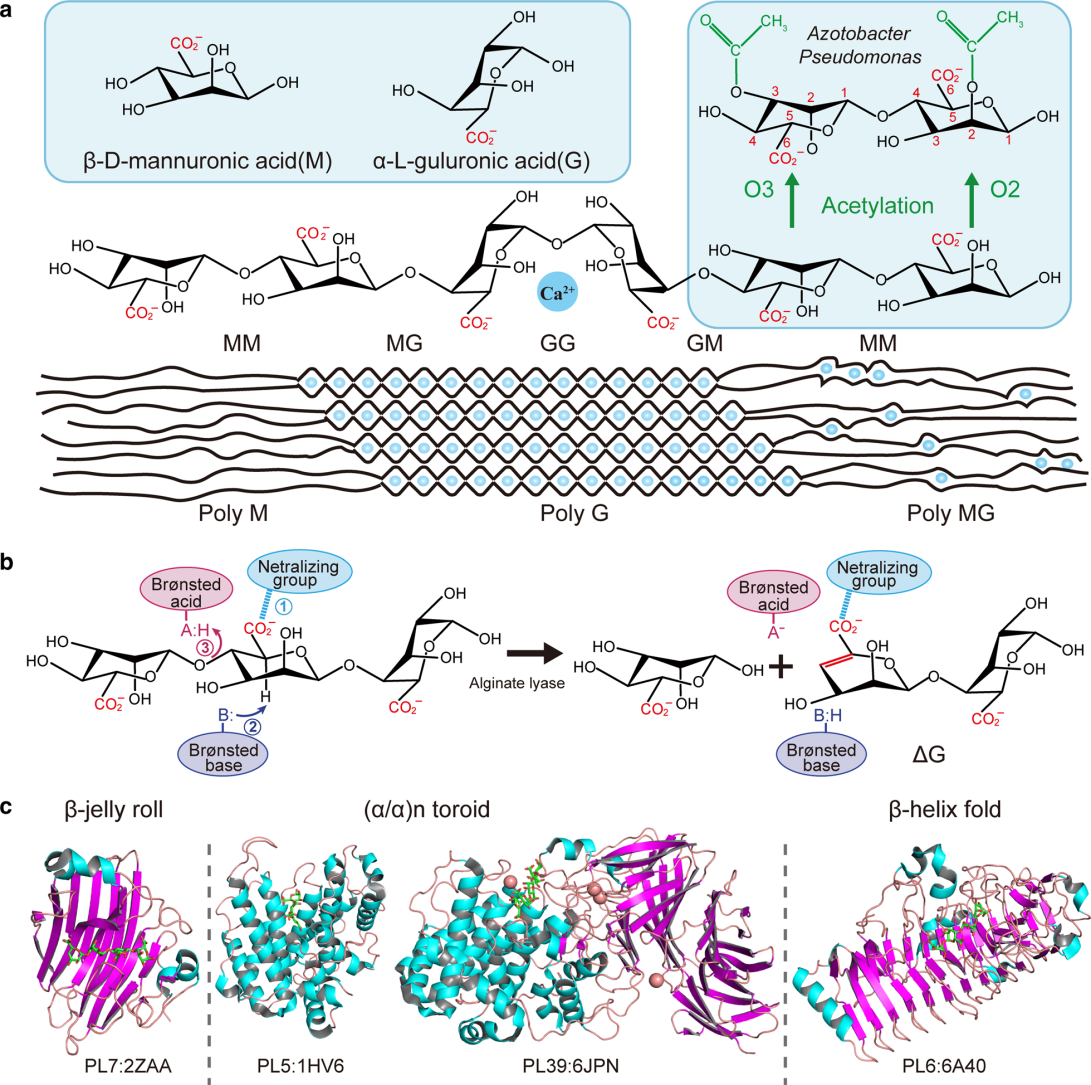
Alginate and alginate lyase. **A** Structures of alginate. Alginates comprise β-d mannuronic acid (M) and α-l-guluronic acid (G), which are arranged in three block-types (MM, GG and MG/GM). In the alginate-producing bacteria *Azotobacter* and *Pseudomonas*, O-2 and/or O-3 of d-mannuronate is acetylated by mannuronan acetylases [15, 16]. **B** Catalytic mechanism of alginate lyase. The negative charge on the carboxylate anion can be neutralised by metal ions or amino acids. The Brønsted acid and base refer to associated amino acids in specific enzymes. **C** Structures of alginate lyases. Left panel, β-jelly roll, PL7 alginate lyase A1-II’ (PDB: 2ZAA) from Sp*hingomonas* sp. A1; middle panel, (α/α)n toroid, PL5 alginate lyase A1-I (PDB: 1HV6) from Sp*hingomonas* sp. A1 and PL39 alginate lyase Dp0100 from *D*. *phaphyphila* (PDB: 6JPN); right panel, β-helix fold, PL6 polyG-specific alginate lyase (PDB: 6A40) from *V*. sp*lendidus*

Alginate is a linear anionic polysaccharide that forms the cell wall in brown macroalgae and some red algae [9]. It is also synthesised by some alginate-producing microorganisms (e.g. *Pseudomonas* [10] and *Azotobacter* [11]). This process is usually under strict regulatory control [12–14]. Unlike alginates present in brown macroalgae, bacterial-derived polymers are often O-acetylated at O-2 and/or O-3 of D-mannuronate, which is catalysed by mannuronate acetylase [15, 16] (Fig. 1A). Acetylation can affect the ion-binding selectivity [17] and water-binding properties of polysaccharides [18].

Alginate consists of β-d mannuronic acid (M) and its C-5-epimers α-l guluronic acid (G) by 1,4-linked glycosidic bond. Alginate consists of 1,4-linked C-5-epimers β-d mannuronic acid (M) and α-l-guluronic acid (G). Both M and G are arranged in at least one of three block types: homopolymeric M-residues (Poly M), homopolymeric G-residues (Poly G), and randomly alternating M- and G-residues (Poly MG; Fig. 1A). The block types and alginate sizes influence the gel formation ability and viscosity of polysaccharides [19]. Due to the variable three-dimensional structures of monomers, the arrangements of the monomeric units are also an important factor affecting the properties of alginates [19]. Moreover, G blocks can form a “buckled” chain conformation and chelate divalent metal ions (Fig. 1A). This structure allows the synthesis of strong but brittle gels, hence the flexibility of polysaccharides is reported to decrease with increasing G content [20].

## Alginate lyases

Alginate lyases are a group of enzymes that catalyse the degradation of alginate via β-elimination of glycosidic bonds. They produce various oligosaccharides with unsaturated uronic acid at the non-reducing end, or 4,5-unsaturated uronic acid monomers mannuronate (ΔManUA) and guluronate (ΔGulUA). Alginate lyases are present in various organisms including marine algae, marine molluscs, fungi, viruses, and a huge number of terrestrial and marine bacteria, of which bacteria are the major producers. Some alginate-producing bacteria, such as *Pseudomonas* *aeruginosa* and *Azotobacter*, are also capable of expressing alginate lyases even though they cannot use alginate as a carbon source [21].

### Classification of alginate lyases

Alginate lyases are categorised into 12 families of polysaccharide lyases in the CAZy database (www.cazy.org): PL5, PL6, PL7, PL14, PL15, PL17, PL18, and the recently identified families PL31, PL32, PL34, PL36 and PL39 [22–24] (Additional file 1: Table S1). Among them, the PL14 family is the mostly widespread and contains alginate lyases from eukaryotes [25–28], bacteria and viruses [29]. Based on their cleavage specificity (i.e. acting on M-rich or G-rich alginates), alginate lyases are classified as M-specific lyases (polyM lyase, EC 4.2.2.3), G-specific lyases (polyG lyase, EC 4.2.2.11), or bifunctional MG lyases (polyMG lyase, EC 4.2.2.-). For example, PL5 family members belong to M-specific lyases, while PL7 family lyases possess different substrate specificities and are able to degrade all types of alginates [30]. Based on their mode of action, all reported members of PL5, PL18, PL31, PL36, and PL39 are endolyases (Additional file 1: Table S1). Among PL7 alginate lyases, most reported PL7 alginate lyases are endolytic except for two members which are exolyases: AlyA5 from *Zobellia* *galactanivorans* Dsij^T^ [31] and VxAly7D from *Vibrio* *xiamenensis* QY104 [32]. PL14 members are also mostly endolyases except for vAL-1 from *Chlorellar* *virus* CVN1 which exhibited endo- and exo-activity [29] and HdAlex from abalone, *Haliotis* *discus* *hannai* which was exolytic [26]. Exolyases are mainly found in oligo-alginate lyase families PL15 and PL17. Among PL15 alginate lyases, A1-IV from Sp*hingomonas* sp. A1 was the first reported exo-type enzyme to be characterised [33, 34]. Notably, PL17 family members Alg17B from BP-2 [35] and AlgL from Sp*hingomonas* sp. MJ3 [36] were shown to be endo- and exo-lytic. The modes of action are listed in Additional file 1: Table S1.

Notably, when heterologously expressed, family PL7 Sp*hingomonas* sp. A1 alginate lyase A1-II’ displayed broad substrate specificity [37]. However, no expression of A1-II’ was observed in cells, even in the presence of alginate, indicating that A1-II’ may be not involved in Sp*hingomonas* sp. A1 alginate metabolism in vivo. Consistent with this, PL7 *Vibrio* *alginolyticus* alginate lyase Aly1 was identified with broad activity on different substrates. However, *aly1* transcript levels were low and not regulated by alginate. This expression pattern is different from other alginate-degrading genes that are up-regulated by alginate (Zhang and Li, unpublished data). Furthermore, deletion of *aly1* did not affect growth or alginate-degrading activity, which further implied that *aly1* may provide only a minor contribution to alginate utilisation in *V*. *alginolyticus* (Zhang and Li, unpublished data). Recently, AusR, the regulator responsible for alginate catabolism in the marine flavobacterium *Z*. *galactanivorans* Dsij^T^, was the first such regulator to be identified [38]. AusR belongs to the GntR family, and can repress the expression of most alginate-utilising genes except for the alginate lyase gene *alyA7*. AlyA7 is proposed to act as a “sentry” enzyme and it is expressed at a basal level even in the absence of alginate. Upon addition of alginate, AlyA7 can release the first oligosaccharide effectors, which can prevent AusR from binding to its targets, thereby triggering a rapid expression of the whole alginate-degrading system [38]. In line with this, *V*. *alginolyticus* Aly1 plays a similar role in alginate catabolism (Zhang and Li, unpublished data). Thus, it is reasonable to hypothesise that Sp*hingomonas* sp. A1 alginate lyase A1-II’ may also be involved in the initial degradation of the polymer.

### Domain organisation of alginate lyases

Besides the catalytic domain, a number of alginate lyases contain auxiliary domains. Carbohydrate-binding modules (CBMs) are the most common auxiliary domain, and they play a key role in alginate degradation. For example, CBM13 in AlyL2 from *Agarivorans* sp. L11 can elevate enzymatic activity and thermostability [39], similar to CBM32 and CMB9 of TsAly7B from *Thalassomonas* sp. LD5 [40]. In agreement with this, truncation of Aly5 non-catalytic regions from *Flammeovirga* sp. strain MY04 not only decreases enzymatic activity and stability, but also causes the accumulation of larger oligomer products, indicating a pivotal role for CBMs in enzyme binding and decomposition of small substrates [41]. AlgH from *Marinimicrobium* sp. H1 [42] and AlyM from *Microbulbifer* sp. CGMCC 14061 [43] exhibit a completely different trait and display higher reactivity and thermal stability following loss of their CBM32 domain. However, the CBM32 domain of AlyQ from *Persicobacter* sp. CCB-QB2 was not involved in the activity of alginate lyase under the tested conditions, but capable of binding to cleaved alginate, which played an important role in the recognition of alginate termini [44]. Because only minimal data are available for the functions of CBMs, their detailed functions and mechanisms related to alginate cleavage and recognition remain unclear. In addition to CBMs, alginate lyases containing tandem catalytic domains that are ubiquitous in many alginate-degrading bacteria [45, 46]. However, the role of individual catalytic domains and their interactions remain unknown.

### Structure of alginate lyases

Except for PL32, PL34 and PL36, the three-dimensional structures of alginate lyases from various families have been determined, and classified into three types: β-jelly roll (PL7, PL14 and PL18), (α/α)n toroid (PL5, PL15, PL17 and PL39), and β-helix fold (PL6 and PL31; Fig. 1C). The β-jelly roll is the most prevalent fold and consists of a pair of antiparallel β-sheets curved nearly 90° in the middle. The inner concave β-strands together with the surrounding loops form a globular or tunnel-shaped core that is particularly important for alginate binding and degradation. Despite sharing a common fold, significant differences are also observed among enzymes with different modes of action, which have been well summarised [47]. Recently, the structure of the novel PL7 alginate lyase AlyC3 from *Psychromonas* sp. C-3 was solved [48]. In this dimeric endo-alginate lyase, dimerisation is an adaptation to seawater salinity.

The (α/α)n toroid fold is a barrel-like architecture consisting of several antiparallel α-helices (Fig. 1C, middle panel). The catalytic cavity includes two outer and inner α-helices layers, and forms a tunnel-like groove. Except for two PL5 folds containing one single (α/α)6 toroid catalytic domain [49], alginate lyases from PL15, PL17 and PL39 are multi-domain enzymes, and structures include one or more additional domains [24, 50, 51]. Notably, although the catalytic groove of the PL39 alginate lyase Dp0100 from the marine thermophile *Defluviitalea* *phaphyphila* resembles that in exolytic lyases of PL15 and PL17 families, analyses of both oligosaccharide product and complex structures with a pentasaccharide suggest that Dp0100 serves as an endolytic alginate lyase [24] (Fig. 1C, middle panel).

The right-handed β-helix class displays two different dimer and monomer catalytic modes. The PL6 alginate lyase AlyGC from *Paraglaciecola* *chathamensis* S18K6T functions as an exolytic and polyG-specific enzyme [52]. It forms a homodimer and each monomer contains a catalytic N-terminal and a C-terminal domain essential for dimerisation and alginate degradation. The conformation of AlyGC is altered when a substrate is bound, thereby leading to only one substrate binding to one active centre [52]. The other three alginate lyases for which structures have been reported, two from PL6 (*Bcel*PL6 from *Bacteroides* *cellulosilyticus* DSM 14838 [53] and AlyF from *Vibrio* sp*lendidus* [54]) and one from PL31 (PsAly from *Paenibacillus* sp. FPU-7 [22]), are endolytic monomeric β-helices, and each harbours one catalytic domain. These alginate lyases catalyse Ca^2+^-assisted β-elimination to degrade substrates, except for the Ca^2+^-independent alginate lyase AlyF [54].

### Catalytic mechanism and sequence characteristics in the active site architecture of alginate lyases

**Fig. 2:**
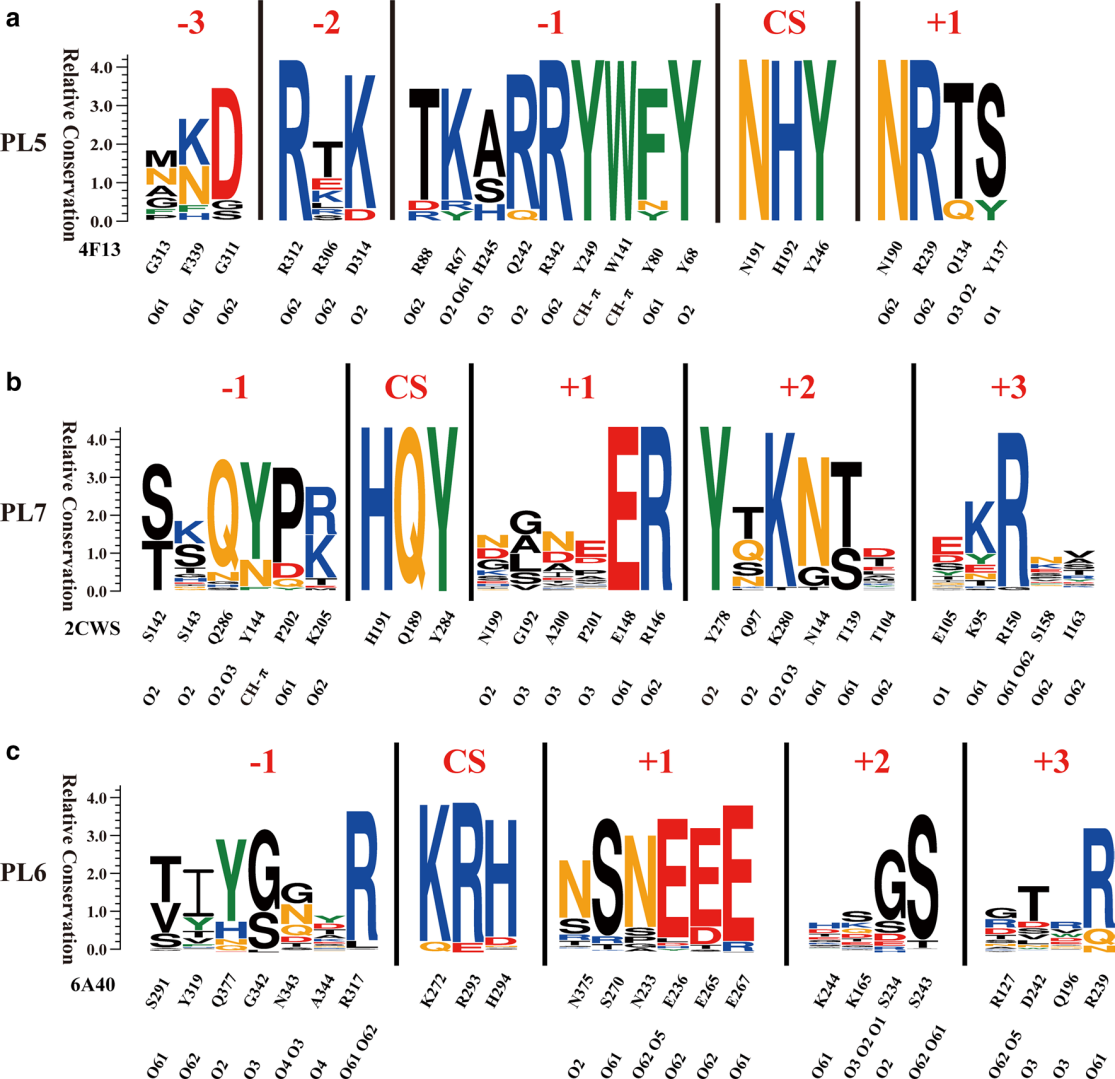
Sequence profiles of different alginate lyase families. Sequence profiles of **A** PL5 (structure template: 4F13) and **B** PL7 (structure template: 2CWS) were obtained by using, respectively, characterised enzymes. The sequence profile of **C** PL6 (structure template: 6A4O) was obtained using all PL6 enzymes in the CAZy database. In each sequence profile, the ordinate indicates the relative degree of conservation, while the abscissa shows the PDB ID of the template structure, as well as the type and sequence number of each amino acid. Each type of amino acid is represented by abbreviated letters with a corresponding colour (KRH, blue; DE, red; NQ, orange; WFY, green; others, black), where the same colour indicates similar physicochemical properties. The locations of ligand atoms interacting with the amino acid residues at each subsite are marked at the bottom, where CS stands for cleavage site. For each protein structure, residues within 5 Å around the active site are displayed and identified as the composition of active site architecture

Although the cleavage mechanisms of alginate lyases have not been fully revealed, a stepwise catalytic process has been suggested [55]. First, the negative charge on the carboxylate anion is quenched via neutralisation by a salt bridge. Next, the proton on C-5 is abstracted by a general based-catalysed reaction, and an enolate intermediate is generated. Finally, electrons are transferred from the carboxyl group, forming a double bond between C-4 and C-5, resulting in the β-elimination of the 4-*O*-glycosidic bond (Fig. 1B). However, alginate lyases belonging to different PL families display different sequence profile characteristics in the active site architecture. For example, in PL5 alginate lyases, which are all M-specific, the conserved catalytic centre sites include asparagine (N), histidine (H) and tyrosine (Y) residues. PL5 A1-III was the first alginate lyases for which the catalytic mechanism was determined, and its catalytic site residue Y246 was proposed to function as both the catalytic base and the acid [49, 56]. Moreover, the sequence profile showed that many other amino acid residues at − 3 to + 1 subsites are also highly conserved, including Arg312, Arg342, Tyr249, Trp141, Asn190 and Arg239 in A1-III (PDB ID: 4F13, template structure; Fig. 2A). Consistent with these A1-III residues [49], our sequence profiling revealed that Arg312 and Arg342 form hydrogen bonds with sugar residues of M-2 O-62 and M-1 O-62, respectively, and CH–π interactions are observed between Tyr249, Trp141 and M-1 (Fig. 2A). In addition, conserved N190 may form hydrogen bonds with M + 1, while Arg239 likely stabilises the negative charge of ionised Y246 [49]. Furthermore, based on our early studies on xylanases [57, 58], variable amino acid residues at the − 3 subsite of the active site architecture might provide a clue to alginate lyase engineering for potential industrial applications.

Sequence profiles of PL7 alginate lyases showed that conserved residues His, Gln and Tyr form the catalytic centre sites, as first demonstrated for A1-II’ (Fig. 2B, AI-II’, PDB ID: 2CWS used as template structure) [59]. Among them, His and Tyr may act as the Brønsted base and the Brønsted acid of the reaction, respectively [48]. Meanwhile, some conserved amino acid residues around the catalytic centre sites were observed, including Glu148, Arg146, Lys280, Tyr278 and Arg150, all candidates for the formation of hydrogen bonds with substrates in A1-II’ (Fig. 2B). In addition, two sets of CH–π interactions were suggested between Tyr284 and Arg146, and between Tyr278 and Arg150 in A1-II’ [59]. Recently, the catalytic process of PL7 alginate lyase AlyC3 was proposed to be similar to that of A1-II’. In addition, conserved residues Arg78 and Gln125 of AlyC3 (corresponding to Arg146 and Gln189 of A1-II’) probably neutralise the negative charge of the carboxylic group at the + 1 subsite, while conserved residues Gln246 and Arg82 (corresponding to Gln286 and Arg150 of A1-II’) are likely involved in substrate binding [48]. However, distinct from other PL7 alginate lyases [59–61], AlyC3 does not require the opening of a loop to bind the substrate [48].

In PL6 alginate lyases, residues in the centre of the active sites are mainly Lys, Arg and His (Fig. 2C, AlyF, PDB ID: 6A40 as template structure). Lys and Arg are suggested to function as the catalytic base and the catalytic acid, respectively [52]. In addition, some conserved residues at − 1 to + 3 subsites also play important roles in alginate degradation, such as Arg317, Asn233, Glu236, Glu265, Glu267 and Arg239 [52–54]. Among them, Asn233, Glu236, Glu265 and Glu267 are required for polar interactions between the substrate and the alginate lyase enzyme. Polar interactions are mediated by Ca^2+^ in AlyGC [52] and *Bcel*PL6 [53] but by water in AlgF [54]. Ca^2+^ may neutralise the carboxyl group of an alginate residue at the + 1 subsite, similar with the role of Arg in PL5 and PL7. Consistent with this, no positively charged residue is observed at the + 1 subsite in PL6 alginate lyases (Fig. 2). Notably, unlike the amino acid residues in the centre of the active sites of PL5 and PL7, which are completely conserved, Lys and His are not observed in all members of PL6, and the absence mainly occurs in the subfamily 3 (Additional file 1: Fig. S1). Further analyses revealed that the catalytic site architecture of superfamily 3 is completely different from that of subfamilies 1 and 2 (Additional file 1: Fig. S1). However, because no PL6 alginate lyase belonging to subfamily 3 has been characterised, the function and active site of PL6-3 remain unknown. Therefore, this distinct catalytic site architecture, which is constructed using enzymes of subfamilies 1 and 2, raises a question whether it is appropriate to classify subfamily 3 into the PL6 family. A more extensive investigation is required to gain insight into the classification and catalytic mechanism of PL6 subfamily 3.

As shown in Additional file 1: Fig. S2, active centre sites of PL15, PL17, PL36, and PL39 families are not completely conserved because only one crystal structure of enzyme in complex with a small oligosaccharide substrate was solved in each family. In PL15 family, His311, Tyr365, and His531 form the catalytic centre sites, and His311 and Tyr365 serve as the catalytic base and acid, respectively [50]. In PL17 family, Tyr258 and Tyr450 are proposed to function as the general acid and base, respectively [51]. However, because residue Tyr450 is not conserved among PL17 members, we cannot exclude that His202 may act as the general base in PL17 members which do not contain Tyr450 in the catalytic centre sites (Additional file 1: Fig. S2). Therefore, more crystal structures of PL17 enzymes are required to fully illustrate the active site residues in catalysis. In PL36 family, residues in the centre of the active sites are conserved, including Tyr185, Arg169, Tyr187, and Lys143 (Additional file 1: Fig. S2). Among them, Lys143 may serve as both the general acid and base, and Tyr185 may play a role in proton transfer [62]. In PL39 alginate lyases, the conserved catalytic centre sites include Tyr239, His405, Asp186, and His187 (Additional file 1: Fig. S2). Tyr239 and His405 likely function as the catalytic acid and base, respectively [24]. In addition, some conserved residues at − 2 to + 3 subsites might also play an important role in alginate degradation. For example, residues Gly340, Tyr135, Ile134, Glu235, Asn404, Arg183 and His185 are probably used for substrate binding (Additional file 1: Fig. S2). Furthermore, more biochemical analyses of alginate lyases and crystal structures of enzymes in complex with ligand are required to fully understand the catalytic mechanism of different alginate lyase families.

## Alginate degradation pathways in vivo

### Three systems for alginate assimilation

**Fig. 3:**
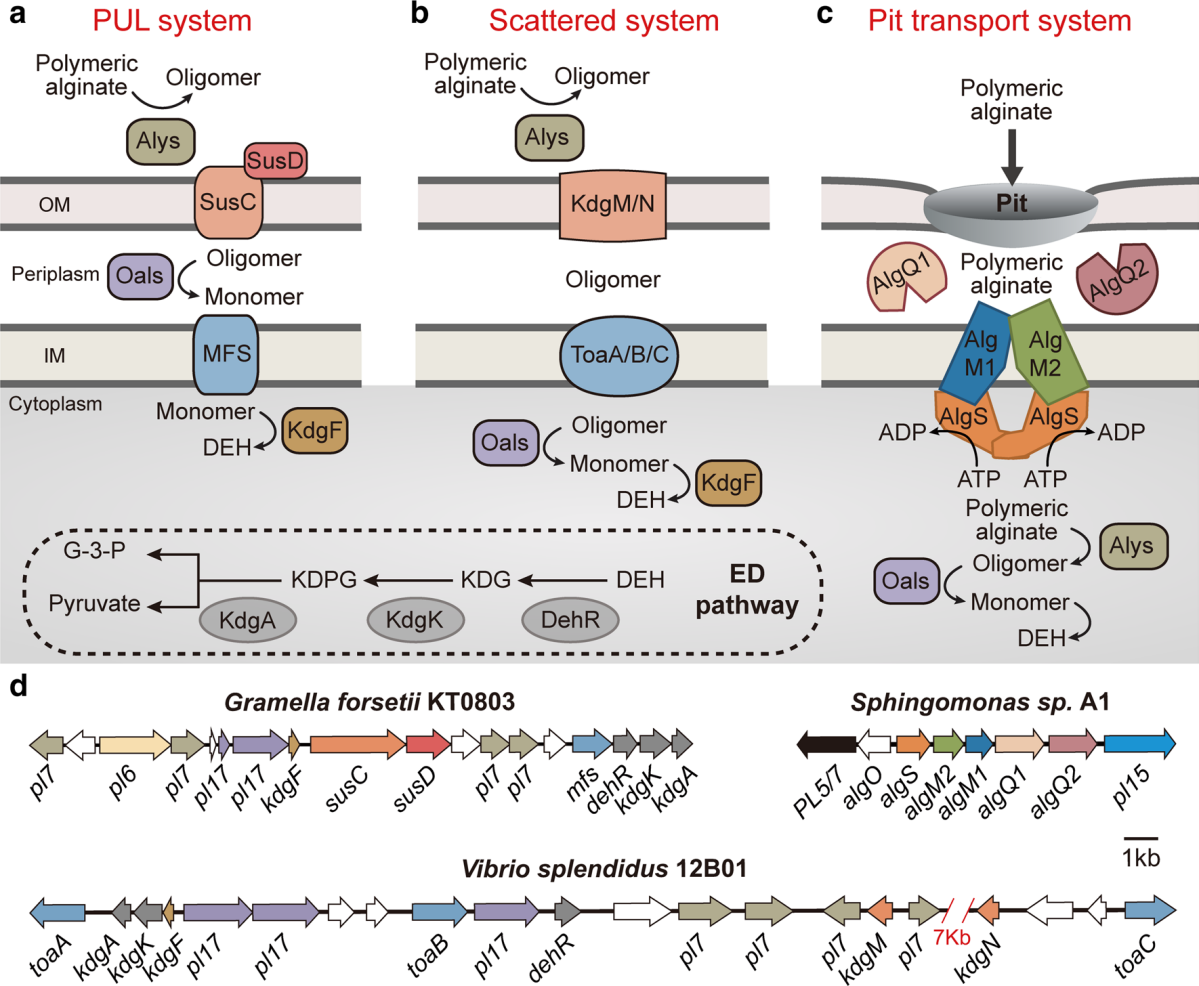
Strategies for alginate utilisation. **A** The PUL system. This system is mainly employed by *Bacteroidetes* and *Gammaproteobacteria* alginate-degrading bacteria. **B** The scattered system. This is mainly present in *Vibrio* bacteria, and genes encoding alginate metabolism enzymes are distributed across the genome. **C** The pit transport system. This system is limited to *Alphaproteobacteria* strain Sp*hingomonas* sp. A1 and its relatives. **D** The location of genes encoding alginate utilisation proteins. The *algO* gene in Sp*hingomonas* sp. A1 encodes a transcriptional regulator

**Fig. 4:**
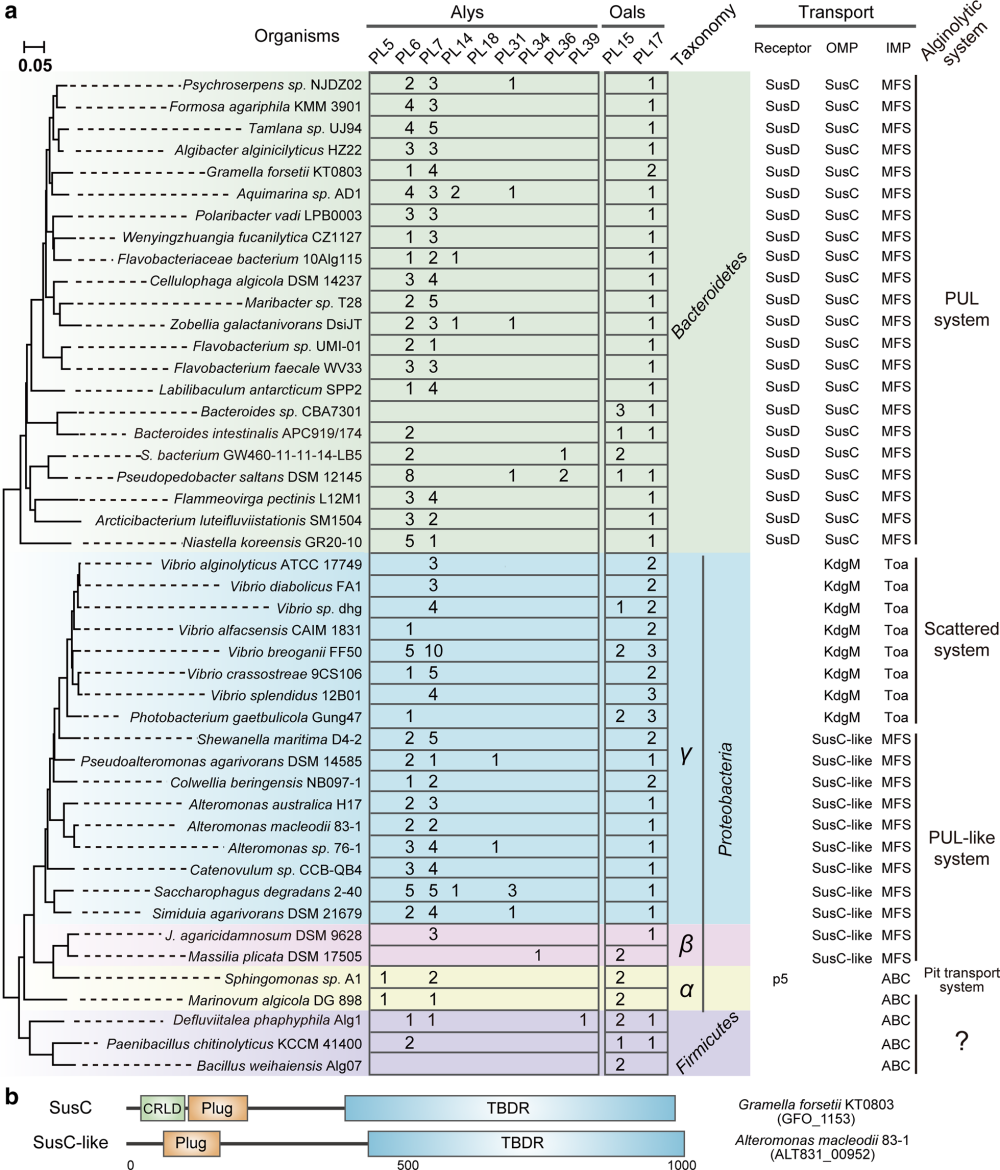
Distribution and abundance of alginate lyases in alginate-utilising microorganisms. **A** Phylogenetic tree of alginate-utilising bacteria with the distribution and abundance of alginate lyases and transporters. In the CAZy database there are ~ 250 species with genome sequence information, and 46 representatives were used to construct the tree based on alignment of 16S rRNA gene sequences. Poly- and oligo-alginate lyases are listed separately and shown as Alys and Oals, respectively. In addition, outer and inner membrane transporters for substrate delivery were analysed and are shown as OMP and IMP, respectively. MFS, major facilitator superfamily; ABC, ATP-binding cassette transporter. **B** Composition of SusC and SusC-like. SusC mainly occurs in *Bacteroidetes* and contains three putative domains: a carboxypeptidase regulatory-like domain (CRLD), a plug domain (an independently folded subunit of the TonB-dependent receptor that acts as a channel gate and blocks the pore when the channel is bound by a carbohydrate substrate), and a TonB-dependent receptor domain (TBDR). SusC-like present in *Gammaproteobacteria* lacks an N-terminal CRLD domain

As mentioned above, knowledge of the functions of alginate lyases has increased dramatically in recent years. However, we have only begun to probe the biological process of alginate decomposition using genetic techniques and omics approaches. To date, three different strategies have been reported for alginate-utilising bacteria: the polysaccharide utilisation loci (PUL) system, the “scattered” system, and the “pit” transport system (Fig. 3). The PUL and scattered systems are defined based on the localisation of alginate degradation-associated genes. Our bioinformatics analysis revealed that the PUL system is common and present in a variety of alginate-utilising bacteria, while the scattered system mainly occurs in *Vibrio* strains. The pit transport system is limited to *Alphaproteobacteria* strain Sp*hingomonas* sp. A1 [63] and its relatives (Fig. 4A).

#### The PUL system

PULs are loci related to polysaccharide utilisation, which were first described by Bjursell et al. [64]. PULs comprise co-regulated genes encoding various substrate-specific carbohydrate-active enzymes, substrate-binding proteins, oligosaccharide transporters, glycan-specific TonB-dependent receptors (SusC proteins), SusD-like substrate-binding proteins (SusD-like), regulators, and hypothetical proteins. First, outer membrane alginate lyases catalyse high molecular weight carbohydrates into oligomers. Once transported into the periplasm by SusC/SusD-like, these oligosaccharide products are converted into monomers by periplasmic alginate lyases. Finally, monomers are delivered into the cytoplasm via major facilitator superfamily (MFS) transporter, and KdgF catalyses the ketonisation of unsaturated monomers into 4-deoxy-L-erythro-5-hexoseulose uronate (DEH) [65]. DEH is stepwise degraded into 2-keto-3-dexy-gluconate (KDG), keto-deoxy-phospho-gluconate (KDPG), glyceraldehyde triphosphate (G-3-P) and pyruvate (Fig. 3A). The occurrence of the *susC*/*susD*-like pair is a hallmark for detection of PULs [66, 67]. Conformational changes of the SusC/SusD-like complex facilitate transport of oligomer products into cells [68]. Moreover, this transporter is not merely restricted to alginate utilisation, but also other major carbohydrates of macroalgae and plants such as mannitol, laminarin, starch and xyloglucan [69].

#### The scattered system

The scattered system comprises genes related to alginate decomposition not co-localised in the same operon, but distributed across different regions of the whole genome. In this system, the alginate-cleaving process is also initiated by extracellular or membrane-attached polysaccharide lyases, which act on the polysaccharide backbone to produce oligomers [70]. After being transported into the cell by outer membrane porin KdgMN and inner membrane symporter ToaABC, these products can be stepwise degraded to 4,5-unsaturated mannuronate or guluronates by specific oligosaccharide lyases [7, 71] (Fig. 3B). Therefore, unlike the PUL system, the scattered system lacks homologs of TonB-dependent receptors and MFS transporters, indicating a different way to import alginate oligomers. Instead, the scattered system employs porin KdgMN for extracellular substrate transport, while it is unclear whether a substrate receptor occurs in this system.

#### The pit transport system

The pit transport system is a completely distinct strategy for alginate decomposition. During alginate assimilation, no extracellular degradation occurs, and alternatively, alginate macromolecules are directly incorporated into the cytoplasm through a pit transport system [33, 63] (Fig. 3C). This system comprises a superchannel on the cell surface, periplasmic binding proteins, and a cytoplasmic membrane-bound ATP-binding cassette (ABC) transporter. When alginate is present, outer membrane alginate-binding proteins, such as flagellin homologs, are responsible for alginate recognition and superchannel formation [72]. Once it enters the cell, alginate is bound to periplasmic binding proteins and delivered to an inner membrane-bound ABC transporter. Finally, cytoplasmic alginate lyases cleave the transferred substrate into monomers. Overall, the most significant differences among the three systems are their distinct recognition and transport components.

### Bacteria with different systems

*Bacteroidetes* are microorganisms that employ the PUL system to degrade various polysaccharides [67, 73, 74]. They are ubiquitous in the marine realm and serve as the major contributor to the consumption of complex organic substrates. Generally, *Bacteroidetes* strains capable of alginate uptake harbour one or more PULs. It was first experimentally verified in *Z*. *galactanivorans*, which harbours two alginate-specific PULs, and both regulons are modulated by alginate [75, 76]. The regulator AusR is responsible for alginate catabolism by repressing the expression of most PUL genes in the absence of alginate [38]. Using quantitative proteomics combined with isotope labelling and subcellular protein fractionation, Kabisch et al. delineated the complete pathway of alginate utilisation in the marine *Bacteroidetes* strain *Gramella* *forsetii* KT0803 (Fig. 3A, D) [73]. A similar process of alginate degradation is also proposed in other *Bacteroidetes*, such as *Flavobacterium* sp. strain UMI-01 [77] and *Formosa* *agariphila* KMM3901^T^ [78], as well as human gut bacterium *Bacteroidetes* *intestinalis* DSM 17393 [79].

Populations of *Proteobacteria* specialised for successive alginate degradation mainly belong to *Gammaproteobacteria*, and two different strategies have been found (Fig. 4A). One is the PUL-like system resembling that in *Bacteroidetes*, and the other is the scattered system mainly present in *Vibrio* species. *Alteromonas* *macleodii*, one of the most important contributors to algal degradation in diverse marine systems [80–83], is a representative of the PUL-like system. It has been shown that *A*. *macleodii* can account for more than 60% of the bacterial community in an alginate-supplemented microcosm [84], and genomic and metabolic variability occurs in different *A*. *macleodii* strains [85]. Among these strains, *A*. *macleodii* 83-1 isolated from an alginate-rich microcosm is usually used as a model strain due to its capability to degrade various algal substrates [83]. However, unlike the “classical” PUL system in *Bacteroidetes*, no *susD*-like gene is found in *Gammaproteobacteria*, and SusC homologs lack an N-terminal domain (carboxypeptidase regulatory-like domain, ~ 70 amino acids, Fig. 4B).

The scattered system is mainly present in *Vibrio* species. Nevertheless, in *Vibrio* species such as *V*. sp*lendidus* [7], most genes responsible for alginate utilisation are clustered in different directions, and are proposed be derived from *Flavobacteriia* [75]. In addition to gene localisation, the lack of homologs of TonB-dependent receptors and MFS transporters indicate that *Vibrio* strains adopt a different method to import alginate oligomers, possibly using outer membrane porin KdgMN and inner membrane symporter ToaABC for oligomer import. Notably, although most oligo-alginate lyases (PL15 and PL17 members) are capable of producing monomers in vitro, the conversion of oligomers to monomers in vivo appears to be accomplished by different oligo-alginate lyases synergistically. For example, although all three oligo-alginate lyases (OalA, OalB and OalC) in *V*. sp*lendidus* strain 12B01 are able to produce monomers in vitro [71], their effects on alginate-dependent growth in vivo are completely different, with no growth when *oalB* is absent, delayed growth when *oalC* is lacking, and wild type (WT)-like growth without *oalA* [7]. Thus, the production of monomers from oligomers is probably achieved by synergy between different oligo-alginate lyases, and similar results were observed in *V*. *alginolyticus* (Zhang and Li, unpublished data).

The pit transport system appears to be limited to *Alphaproteobacteria* strain Sp*hingomonas* sp. A1 and its relatives. Unlike the PUL and scatter systems, strain A1 imports alginate into the periplasm without prior depolymerisation. The outer membrane protein P5, homologous to bacterial flagellin, acts as a receptor for alginate transport [72]. Moreover, the function of flagellin P5 binding to alginate is proposed to be a universal property of flagellins since the *Escherichia* *coli* flagellin FliC is also capable of alginate interaction in vitro [72]. In addition to P5, another surface protein (Algp7) is involved in alginate binding [86]. Algp7 is homologous to lipoproteins, but lacks a lipid moiety. Instead, it harbours a metallopeptidase motif, and has a domain homologous to the M75 peptidase motif-containing protein EfeO, a component of a ferrous ion transporter [87]. Algp7 has an affinity for metal ions, hence it may deprive alginate/metal complexes of metal ions [88]. When alginate enters the cell, it is bound by the periplasmic solute-binding proteins AlgQ1 or AlgQ2, and transferred to the ABC transporter AlgM1M2SS, a heterotetramer of AlgM1, AlgM2 and AlgS [89–91]) (Fig. 3C, D). An overview of the pit transport system has been intensively reviewed recently [63].

Besides *Bacteroidetes* and *Proteobacteria*, several microorganisms belonging to *Firmicutes* are also capable of alginate metabolism. For example, the thermophilic marine strain *D*. *phaphyphila* harbours a complete pathway for alginate utilisation, and can directly convert alginate to ethanol [92, 93]. In addition, an alginolytic pathway is also suggested in the marine strain *Bacillus* *weihaiensis* [94]. During algal decomposition, *B*. *weihaiensis* exhibits enzymatic activity toward alginate and laminarin substrates. However, the prioritisation of alginate over laminarin is different from that in *A*. *macleodii* 83–1, with initial laminarin consumption followed by simultaneous alginate and pectin degradation [94, 95]. The difference probably results from different ecological niches. Bioinformatic analysis reveals that these *Firmicutes* bacteria contain scattered genes for alginate degradation (similar to the scatter system), but use an ABC transporter for oligomer uptake, similar to the pit transport system (Fig. 4A). Further genetic investigation is required to fully establish the alginate metabolic pathway in *Firmicutes*.

### Different ecophysiological types of alginate utilisation

Within strains of the common marine *Vibrionaceae* bacteria, a huge diversity of alginate-decomposing enzymes have been identified, and many of these strains are associated with animals [96]. *Vibrionaceae* species co-occurring in the same environmental samples display differences in alginate degradation pathways [97]. This differentiation may result from horizontal gene transfer, leading to adaptive radiation, thereby mitigating competitive exclusion and fine-scale resource partitioning of alginate [97]. On this basis, Hehemann et al. defined three different ecophysiological types for alginate utilisation: pioneer, scavenger, and harvest [97]. Pioneers possess extracellular polysaccharide and oligosaccharide lyases, and are able to secrete alginate lyases that cleave insoluble alginate polymers into soluble polymers and oligomers. These products are subsequently utilised by harvest and scavenger classes. Scavengers that only contain oligosaccharide lyases cannot decompose alginate directly, but can utilise oligomers. In contrast, harvest organisms are selfish and have membrane-anchored poly- and oligo-alginate lyases, and can therefore both degrade polymers and use oligomers produced by pioneers [97]. The scattered system in *Vibrio* strains may be more beneficial for adapting to different niches, resulting in different ecophysiological types supporting coexistence.

Different strategies for alginate degradation are also observed in microorganisms belonging to different phyla. For example, a biogeographical study of bacterial polysaccharide degradation in the Atlantic Ocean revealed that pioneers and scavengers predominantly belong to the *Alteromonadaceae* and SAR11 clades, respectively, while harvest strains mainly belong to *Bacteroidetes*, *Gammaproteobacteria* and *Planctomycetes* [98]. Consistent with this, marine *Bacteroidetes* member *G*. *forsetii* functions as a harvest strain, similar to members of gut *Bacteroidetes* [99]. However, in California coastal seawater, *Alteromonadaceae* *A*. *macleodii* strain 83-1 appears to be selfish since it cannot benefit non-alginolytic microorganisms by cross-feeding on alginate degradation or other metabolic products when alginate particles are supplied [100]. Therefore, bacterial hierarchy for polysaccharide utilisation may be largely dependent on bacterial community composition and environmental factors, such as polysaccharide types.

## Applications and prospects of alginate lyases and associated metabolic pathways

### Alginate lyases for alginate oligosaccharide preparation

**Fig. 5:**
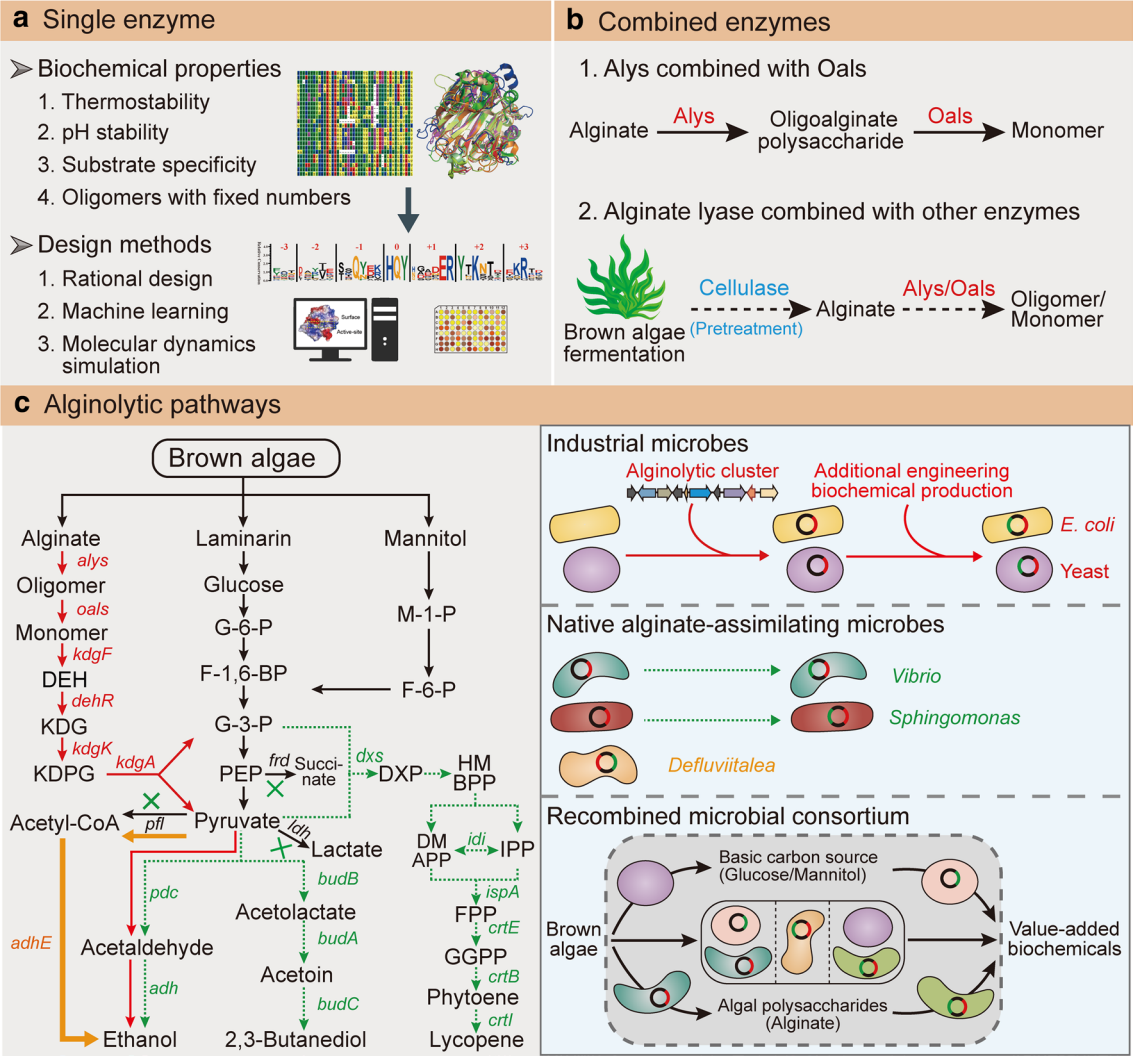
Application of alginate lyases and associated metabolic pathways. The schematic diagram shows the application of single (**A**) and combined (**B**) enzymes. **C** Schematic pathways for the production of bioethanol and other value-added biochemicals. A workflow for ethanol production in industrial microbes (*E*. *coli* and *S*. *cerevisiae*) is shown as red solid arrows. In *S*. *cerevisiae*, the eukaryotic DEH transporter DHT1 is introduced for DEH entry. The workflow for bioethanol production in native alginate-utilising bacteria Sp*hingomonas* sp. A1 and *Vibrio* sp. dhg is shown as green dashed arrows. Besides ethanol production, *Vibrio* sp. dhg was also engineered to synthesise 2,3-butanediol and lycopene. Alginate-utilising strain *D*. *phaphyphila* harbours a distinct pathway and stepwise converts pyruvate into acetyl-CoA and ethanol, which is shown as orange solid arrows. In *E*. *coli* and *Vibrio* sp. dhg, endogenous genes including *frd* encoding fumarate reductase, *ldh* encoding lactate dehydrogenase, and *pfl* encoding pyruvate-formate lyase, were deleted to block by-product synthesis, shown as green crosses. In Sp*hingomonas* sp. A1, only lactate formation is inhibited by deletion of *ldh* gene

Alginate oligosaccharides (AOS) possess attractive biological properties, especially versatility and beneficial effects on human health. Their bioactivities have been summarised comprehensively by Liu et al. including prebiotic, antitumor, antihypertensive, anti-diabetic, antimicrobial, antioxidant, anticoagulant, and immunomodulatory activities [3]. Traditional methods for AOS production require strong acid and alkaline [101], which may cause environmental pollution. In contrast, enzymatic methods are more environmentally friendly. However, except for one commercialised alginate lyase (CAS number: 9024-15-1, Sigma-Aldrich), most alginolytic enzymes have been studied at the laboratory level, and show great potential at high pH and impressive heat stability (Fig. 5A). For example, alginate lyase AMOR_PL7A from a hot vent in the Arctic Mid-Ocean ridge displayed stable polyM-specific endolytic activity over a wide temperature range (60–80 °C), pH (4.7–7.8) and salinity (0–2 M) [102]. Alginate lyase NitAly from marine strain *Nitratiruptor* sp. SB155-2 exhibits highest activity at 70 °C [103], while PL36 family alginate lyase Aly36B from *Chitiophaga* sp. MD30 [62] and Aly08 from *Vibrio* sp. SY01 [104] are alkaline-stable, with optimal pH values of 9.0 and 8.35, respectively. In addition to enzymes with high pH and thermostability, some alginate lyases also display positive traits for the preparation of alginate oligomers with different degrees of polymerisation. For example, Alg2A from *Flavobacterium* sp. S20 can produce high yields of oligosaccharides with a high degree of polymerisation (e.g. penta-, hexa- and hepta-saccharides) [105].

Despite increasing numbers of identified enzymes for the production of marine polysaccharides (e.g. alginate), our understanding of marine enzymatic activity and mechanisms is lacking compared with that of terrestrial enzymes. However, rapid growth in the number of marine-derived enzymes and “multi-omics” data make it possible to obtain potential commercial enzymes for macroalgae degradation by not only identifying new enzymes with excellent capability from natural environments, but also rational design combined with machine and deep learning approaches. Using powerful computational approaches, alginate lyases may be customised to produce oligomers with fixed numbers of units. Likewise, multifunctional alginate lyases with completely different catalytic functions can also be designed. For example, PL17 alginate lyase Smlt2602 from *Stenotrophomonas* *maltophilia* K279a exhibits a unique property and provides an important clue to developing multifunctional enzymes [106]. When its His208 residue was changed to Phe208, the resulting H208F mutant displayed significant catalytic activity toward glucuronan, a non-alginate-based substrate [106]. This was also the first alginate lyase capable of exolytic glucuronan activity. However, the mechanism leading to the digestive turnover of two different substrates is unclear, and crystal structures of Smlt2602 and H208F bound to their respective substrates are required.

### Combined enzymes for AOS preparation

Besides single enzymes, different types of alginate lyases can produce AOS synergistically (Fig. 5B). For example, combining alginate lyase AlyPB1 with oligo-alginate lyase AlyPB2 is more efficient for AOS formation due to synergistic digestion [107]. In addition to combining alginate lyases, a mixture of different enzyme classes may be more favourable. Li et al. reported a rapid and efficient method for producing AOS directly from brown algae via tandem usage of cellulase and alginate lyase [108]. First, dried algae powder is digested by cellulase to release the fermentable sugars glucose and mannitol. Subsequently, the yeast strain *Yarrowia* *lipolytica* expressing an extracellular alginate lyase is fermented with cellulase-pretreated algae medium. During fermentation, glucose and mannitol are consumed while AOS are released into the supernatant [108]. The improvement in brown algae-degrading efficiency in vitro may also be compatible with terrestrial carbohydrate materials [109], hence it may be possible to tailor-make a set of substrate-associated enzymes. This approach is very common in the enzymatic hydrolysis of many agricultural residues such as rice straw [110], wheat straw [111], corn stover [112] and sugarcane bagasse [113]. It combines a biochemical approach with mathematical analysis to develop an efficient and mixed customised enzymatic system.

### Applications and prospects of alginolytic pathways

#### Engineering of industrial microbes

Because common industrial microorganisms cannot utilise alginate due to the lack of an alginate metabolic pathway, it was impossible to achieve a complete conversion from brown algae to ethanol until 2012 when Wargacki et al. [7] engineered an appropriate microbial platform (Fig. 5C). In this platform, *E*. *coli* is used as a host, and alginate lyase SM0524Aly from *Pseudoalteromonas* sp. SM0524 is secreted by an autotransporter. In addition, a large gene cluster encoding alginate metabolism enzymes in *V*. sp*lendidus* 12B01 was introduced, accompanied by other auxiliary candidates for complete alginate degradation. Finally, to produce ethanol, a heterologous pathway containing a pyruvate decarboxylase (Pdc) and an alcohol dehydrogenase B (AdhB) was integrated into the *E*. *coli* chromosome, while endogenous *E*. *coli* genes responsible for the formation of fermentative by-products were deleted. Using the platform, the fermentative yield with alginate, mannitol and glucan reached 0.28 g ethanol per g dry brown algae, over 80% of the maximum theoretical yield [7]. Subsequently, Yoshikuni and co-workers developed a more stable system through recombinase-assisted genome engineering (RAGE) to produce ethanol directly from brown algae [114]. Using this system, a heterologous gene cluster for alginate metabolism was integrated into the *E*. *coli* chromosome, leading to a ~ 40% improvement in ethanol yield compared with the plasmid-based counterpart. Meanwhile, the integrated alginate pathway displayed stable phenotypes over 50 generations, with *E*. *coli* cells reaching slightly higher productivities than the original inocula [114].

In 2014, Enquist-Newman et al. attempted to produce ethanol in a safer microbe, *Saccharomyces* *cerevisiae* [8]. Apart from an active transporter for DEH, genes originating from bacteria were codon-optimised for the stepwise conversion of DEH to pyruvate in *S*. *cerevisiae*, including *dheR*, *kdgK*, and KDPG aldolase gene *kdgA*. To solve the problem of DEH import, eukaryotic DEH transporter DHT1 from the deuteromycete *Asteromyces* *cruciatus* was identified to be critical for DEH entry. Additionally, the native mannitol catabolism genes encoding mannitol-2-dehydrogenase and a mannitol transporter were overexpressed to efficiently metabolise mannitol. Using this platform, the ethanol yield supplemented with DEH and mannitol was up to 4.6% v/v (Table 1), approximately 83% of the maximum theoretical yield [8]. Later, an alginate-utilising *S*. *cerevisiae* recombinant strain was constructed by chromosomal integration of genes encoding alginate degradation proteins, and at the same time, the mannitol-utilising capacity was enhanced by prolonged growth with mannitol as the sole carbon source [115]. This engineered *S*. *cerevisiae* strain was capable of directly producing bioethanol from mannitol and alginate, although ethanol yields were not very high (8.8 g g^−1^ mixture of alginate and mannitol, ~ 32% of the maximum theoretical yield; Table 1). Meanwhile, Matsuoka et al. constructed two engineered *S*. *cerevisiae* strains and further improved their aerobic growth in DEH liquid medium through adaptive evolution [116]. Among different mutations in both evolved strains, an E17G mutation in the codon-optimised NAD(P)H-dependent DEH reductase was proposed to be essential for both enhanced activity of DEH reductase and aerobic growth [116].

**Table 1:** The ethanol yield of different microorganisms

Microorganism	Characteristics	Major sugars utilised	Pretreatment	Productivity of ethanol	Reference
*Escherichia* *coli* BAL1611	Engineered strain	Mannitol, alginate, glucose	No pretreatment	0.28 g g^−1^ powder of kelp	[7]
*Saccharomyces* *cerevisiae* BAL3215	Engineered strain	Mannitol, DEH	N/A	0.22 g g^−1^ DEH and mannitol (1:2 molar ratio)	[8]
*Saccharomyces* *cerevisiae* AM1	Engineered strain	Mannitol, DEH	N/A	0.15 g g-1 alginate and mannitol (1:2 molar ratio)	[125]
Saccharomyces cerevisiae MK5622	Engineered strain	DEH	N/A	0.12 g g^−1^ DEH	[116]
Sp*hingomonas* sp. A1	Engineered strain	Alginate	N/A	0.26 g g^−1^ alginate	[117]
*Vibrio* sp. dhg	Engineered strain	Mannitol, alginate	No pretreatment	0.35 g g^−1^ powder of kelp	[119]
*Defluviitalea* *phaphyphila* Alg1	Natural strain	Mannitol, alginate, laminarin	No pretreatment	0.25 g g^−1^ powder of kelp	[126]
*Clostridium* *phytofermentans*	Natural strain	Alginate	Acid pretreatment	0.05 g g^−1^ alginate	[120]

#### Engineering of native alginate-assimilating microbes

Besides the engineered alginate pathway in industrial microbes, some alginate-utilising bacteria per se show great potential for the bioconversion of brown algae. Sp*hingomonas* sp. A1 was the first engineered native microbial platform to produce ethanol from alginate [117]. Two heterogeneous genes encoding Pdc and Adh from *Zymomonas* *mobilis* were introduced and overexpressed in Sp*hingomonas* sp. A1, and the lactate by-product synthesis pathway was blocked to increase ethanol productivity (resulting in Sp*hingomonas* sp. A1 ethanologenic strain MK3353). After a 3-day incubation, the ethanol yield was 0.26 g g^−1^ alginate. However, because MK3353 is unable to grow under anaerobic conditions, the tricarboxylic acid (TCA) cycle can consume pyruvate, and thus compete with ethanol production [117]. Later, Fujii et al. found that toxic by-products accumulated and inhibited the growth of MK3353 during ethanol fermentation from alginate, which was partially overcome by pH adjustment [118].

In 2019, the fast-growing *Vibrio* sp. dhg strain was engineered, and reported to be capable of degrading alginate [119]. Bioinformatic analysis revealed that *Vibrio* sp. dhg contained both alginate and mannitol metabolic pathways. Based on this, different strategies were adopted to generate different products from brown algae sugar mixtures, such as ethanol, 2,3-butanediol and lycopene [119] (Fig. 5C, left panel). In this platform, transcription and translation related to the degradation of polysaccharide substrates can be controlled, and many genetic circuits used in *E*. *coli* are available [119], which together make this strain promising for further applications. Notably, engineered *Vibrio* sp. dhg displayed the highest ethanol yield (0.35 g g^−1^ kelp) among all reported microorganisms that converted alginate to ethanol (Table 1).

The *Firmicutes* bacterium *D*. *phaphyphila* isolated from coastal sediment may be the first characterised microorganism capable of directly converting alginate to ethanol [92, 93]. In its genome, a complete ethanol synthesis pathway is present, including many genes related to alginate degradation, mannitol metabolism, Embden–Meyerhof–Parnas pathway (EMP), Entner–Doudoroff (ED) pathway, and aldehyde dehydrogenases (Fig. 5C, left panel, [92, 93]). Therefore, unlike other microorganisms in which it is hard to control redox homeostasis from metabolism assimilation under anaerobic conditions, *D*. *phaphyphila* Alg1 can balance the reducing equivalents in fermenting brown algae via two complementary pathways (alginate and mannitol metabolism pathways) [92, 93]. However, it is not clear how this strain regulates the reducing equivalents precisely. In addition, this strain has at least two inherent advantages for ethanol production: (i) direct utilisation of pretreated brown algae with a comparatively high ethanol yield of 0.25 g g^−1^ kelp, and (ii) a thermostable alginolytic system up to 60 °C [92, 93]. Recently, the anaerobic *Firmicutes* strain *Clostridium* *phytofermentans* was also found to convert alginate directly from brown algae directly to ethanol [120]. However, genetic tools have not been developed for these microorganisms. Therefore, effective genetic tools may be required to understand and consequently improve alginate and related metabolic pathways, and to elevate the yields of ethanol and other high-value biochemical products.

#### Microbial consortia

In addition to engineering single microbes for the production of ethanol and other biochemicals, a co-culture platform was developed for producing ethanol directly from brown macroalgae [121]. In this system, two types of engineered *S*. *cerevisiae* strains, one for mannitol and alginate assimilation and the other for cellulase synthesis, were supplied to ferment macroalgae *Ecklonia* *kurome*, and ethanol production reached 0.17 g g^−1^ mixture of alginate and mannitol [121]. This approach is the only reported system to apply a microbial consortium for alginate utilisation, and it has advantages for efficient utilisation of varying amounts of carbohydrate components in brown macroalgae through adjusting the ratio of co-culturing yeasts. Although few data about modular microbial co-cultivating approaches are available for bioethanol production from macroalgae, a variety of chemical compounds have been produced from terrestrial carbohydrates using microbial consortia for metabolic engineering [122, 123]. These successful cases will certainly provide valuable hints for further improvements in the bioconversion of macroalgae-derived carbohydrates.

## Conclusions and perspectives

Alginates, major components of brown macroalgae, have attracted much attention for biorefining. Exciting developments include the discovery of alginate lyases with excellent biochemical properties, and the bioconversion of ethanol and other commercial chemicals from alginate, albeit on a laboratory scale (Fig. 5). Compared with long-term research on enzymes and metabolic pathways for the utilisation of terrestrial carbohydrates, understanding enzymes (e.g. alginate lyases) related to macroalgae degradation is just beginning. There are many challenges related to alginate lyases and associated metabolic pathways before industrial application. First, the variable structures of alginate are not fully unlocked, making the understanding of enzymatic action mechanisms more complicated. Furthermore, the catalytic patterns of alginate lyases are not fully elucidated. Except for some PL families with crystal structures, only a limited number of alginate lyase enzyme topologies have been determined, and none are available for recently discovered families such as PL32 and PL34.

Regarding the application of alginate and brown algae, the potential of alginate lyases and related metabolic pathways for biorefining has been demonstrated. However, establishment of a universal approach for industrial bioconversion of brown algae has not been reported. This may reflect comparatively little research on alginate pathways. As mentioned above, although numerous alginate lyases are capable of poly- and/or oligo-alginate degradation in vitro, their roles in vivo remain unknown due to a lack of operable genetic methods for some native alginate-degrading microbes (e.g. *D*. *phaphyphila* [93] and *C*. *phytofermentans* [120]), or detailed metabolic and regulation models for ubiquitous microbes, especially those with the scattered system (e.g. *Vibrio* [7, 119]). Therefore, these detailed functional investigations will allow the engineering of effective native ethanol-producing systems, as well as the modulation of alginate and other associated metabolic pathways to balance fermentation. In addition, with advancements in omics approaches and an increased understanding of alginate utilisation, microbiome engineering, a new concept referred to as “leveraging fundamental scientific principles and quantitative design to create microbiomes that perform desired functions” [124], might be also applied for bioconversion of brown macroalgae in the future.

## Supplementary Information

**Additional file 1** Figure S1. Sequence profiles of different PL6 subfamilies. Figure S2. Sequence profiles of PL15, PL17, PL36 and PL39 families. Table S1 Characteristics of alginate lyases.

## Data Availability

Not applicable.

## Abbreviations

M: β-D Mannuronic acid
G: α-L Guluronic acid
ΔManUA: 4,5-Unsaturated uronic acid monomers mannuronate
ΔGulUA: 4,5-Unsaturated uronic acid monomers glucuronate
CBM: Carbohydrate-binding modules
PL: Polysaccharide lyase
PUL: Polysaccharide utilisation loci
DEH: 4-Deoxy-l-erythro-5-hexoseulose urinate
KDG: 2-Keto-3-dexy-gluconate
KDPG: Keto-deoxy-phospho-gluconate
G-3-P: Glyceraldehyde triphosphate
ABC: ATP-binding cassette
AOS: Alginate oligosaccharides
RAGE: Recombinase-assisted genome engineering
