# Supplementary material for: Bacterial alginate metabolism: an important pathway for bioconversion of brown algae
Source: Biotechnol Biofuels. 2021 Jul 18;14:158. doi: 10.1186/s13068-021-02007-8 (PMC8286568; doi:10.1186/s13068-021-02007-8)
Supplement: Supplementary file 1 — Additional file 1 Figure S1. Sequence profiles of different PL6 subfamilies. Figure S2. Sequence profiles of PL15, PL17, PL36 and PL39 families. Table S1 Characteristics of alginate lyases. [file 13068_2021_2007_MOESM1_ESM.docx]

**Bacterial alginate metabolism: An important pathway for bioconversion of brown algae**

Lanzeng Zhang^a^, Xue Li^a^, Xiyue Zhang^a^, Yingjie Li^a*^, Lushan Wang^a^

^a^State Key Laboratory of Microbial Technology, Shandong University, Qingdao 266237 China

*Corresponding author: Yingjie Li, email: [yingjie.li@sdu.edu.cn](mailto:yingjie.li@sdu.edu.cn)

**
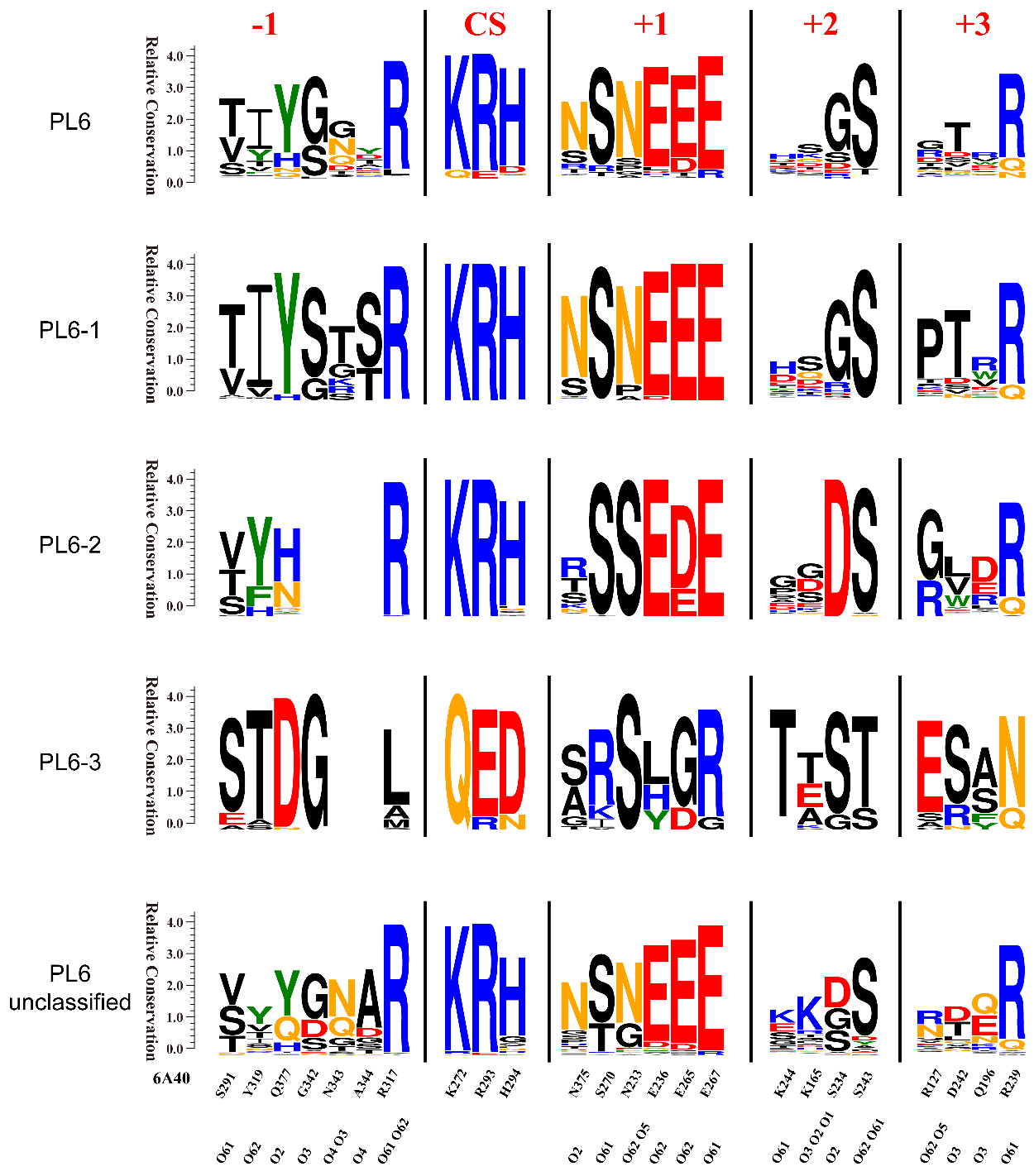
**

**Figure S1. Sequence profiles of different PL6 subfamilies.** The sequence profile of PL6 was obtained using all PL6 enzymes in the CAZy database (structure template: 6A4O). Sequence profiles of PL6-1, PL6-2 and PL6-3 were obtained using their respective enzymes in the CAZy database. Sequence profiles of unclassified PL6 enzymes were obtained using unclassified PL6 enzymes in the CAZy database. In each sequence profile, the ordinate indicates the relative degree of conservation, while the abscissa represents the PDB ID of the template structure, as well as the type and sequence number of each amino acid. Each type of amino acid is represented by abbreviated letters with a corresponding colour (KRH, blue; DE, red; NQ, orange; WFY, green; others, black), where the same colour indicates similar physicochemical properties. The locations of ligand atoms interacting with the amino acid residues at each subsite are marked at the bottom, where CS stands for cleavage site. For each protein structure, residues within 5 Å around the active site are displayed and identified as the composition of active site architecture.


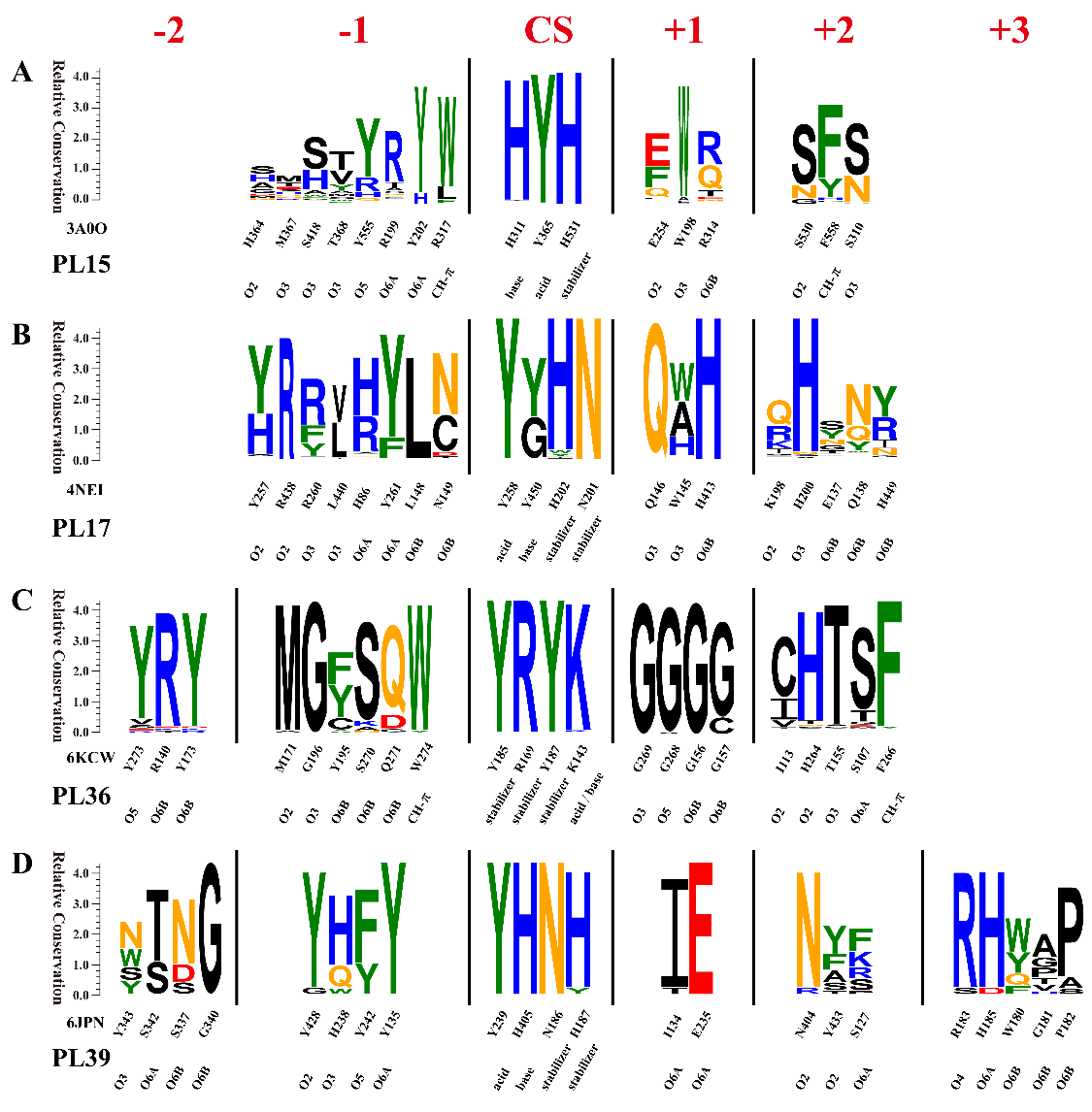


**Figure S2. Sequence profiles of PL15, PL17, PL36 and PL39 families.** Sequence profiles of (A) PL15 (structure template: 3A0O), (B) PL17 (structure template: 4NEI), (C) PL36 (structure template: 6KCW) and (D) PL39 (structure template: 6JPN) were obtained by using respectively enzymes in the CAZy database. In each sequence profile, the ordinate indicates the relative degree of conservation, while the abscissa shows the PDB ID of the template structure, as well as the type and sequence number of each amino acid. Each type of amino acid is represented by abbreviated letters with a corresponding colour (KRH, blue; DE, red; NQ, orange; WFY, green; others, black), where the same colour indicates similar physicochemical properties. The locations of ligand atoms interacting with the amino acid residues at each subsite are marked at the bottom, where CS stands for cleavage site. For each protein structure, residues within 5 Å around the active site are displayed and identified as the composition of active site architecture.

**Table S1 Characteristics of alginate lyases**

| **CAZy family** | **Number of identified alginate lyase** | **Action mode** | **Number of solved enzyme structure** | **Structure type** | **Distribution** |
| --- | --- | --- | --- | --- | --- |
| PL5 | 13 | Endo | 2 | (α/α)n toroid | Bacteria |
| PL6 | 22 | Endo/exo | 3 | β-helix fold | Bacteria |
| PL7 | 44 | Endo^1^ | 11 | β-jelly roll | Bacteria, Eukaryota, Viruses |
| PL14 | 8 | Endo^2^ | 2 | β-jelly roll | Bacteria, Archaea, Eukaryota, Viruses |
| PL15 | 9 | Exo | 2 | (α/α)n toroid | Bacteria |
| PL17 | 7 | Exo^3^ | 1 | (α/α)n toroid | Bacteria |
| PL18 | 4 | Endo | 2 | β-jelly roll | Bacteria |
| PL31 | 3 | Endo | 1 | β-helix fold | Bacteria |
| PL32 | 1 | - | - | - | Bacteria |
| PL34 | 1 | - | - | - | Bacteria |
| PL36 | 2 | Endo | 1 | β-jelly roll | Bacteria, Eukaryota |
| PL39 | 1 | Endo | 1 | (α/α)n toroid | Bacteria |

^1^Most reported PL7 alginate lyases are endolytic except for two members of PL7 family which are exolyase, including AlyA5 from *Zobellia galactanivorans DsijT* [[1](#_ENREF_1)] and VxAly7D from *Vibrio xiamenensis* QY104 [[2](#_ENREF_2)].

^2^Except for vAL-1 from *Chlorellar virus* CVN1 which exhibited endo- and exo-activity [[3](#_ENREF_3)] and HdAlex from abalone, *Haliotis discus hannai,* which was exolytic [[4](#_ENREF_4)].

^3^Except for Alg17B from BP-2 [[5](#_ENREF_5)] and AlgL from *Sphingomonas* sp. MJ3 [[6](#_ENREF_6)] which were shown to be endo- and exo-lytic.

**References**

1. Thomas F, Lundqvist LCE, Jam M, Jeudy A, Barbeyron T, Sandstrom C, Michel G, Czjzek M. Comparative characterization of two marine alginate lyases from *Zobellia galactanivorans* reveals distinct modes of action and exquisite adaptation to their natural substrate. Journal of Biological Chemistry. 2013;288:23021-23037.
2. Tang LY, Wang Y, Gao S, Wu H, Wang DN, Yu WG, Han F. Biochemical characteristics and molecular mechanism of an exo-type alginate lyase VxAly7D and its use for the preparation of unsaturated monosaccharides. Biotechnol Biofuels. 2020. DOI: 10.1186/s13068-020-01738-4
3. Ogura K, Yamasaki M, Yamada T, Mikami B, Hashimoto W, Murata K. Crystal structure of family 14 polysaccharide lyase with pH-dependent modes of action. Journal of Biological Chemistry. 2009;284:35572-35579.
4. Suzuki H, Suzuki K, Inoue A, Ojima T. A novel oligoalginate lyase from abalone, *Haliotis discus hannai*, that releases disaccharide from alginate polymer in an exolytic manner. Carbohydrate Research. 2006;341:1809-1819.
5. Huang GY, Wen SH, Liao SM, Wang QZ, Pan SH, Zhang RC, Lei F, Liao W, Feng J, Huang SS. Characterization of a bifunctional alginate lyase as a new member of the polysaccharide lyase family 17 from a marine strain BP-2. Biotechnology Letters. 2019;41:1187-1200.
6. Park HH, Kam N, Lee EY, Kim HS. Cloning and characterization of a novel oligoalginate lyase from a newly isolated bacterium *Sphingomonas* sp. MJ-3. Marine Biotechnology. 2012;14:189-202.
